# Primary care physicians’ role perception and self-reported performance in glaucoma care: a survey study

**DOI:** 10.1186/s13104-015-1770-z

**Published:** 2015-12-12

**Authors:** Albina Rotshtein, Khaled Karkabi, Orna Geyer, Orit Cohen Castel

**Affiliations:** Department of Family Medicine, Clalit Health Services, Haifa and Western Galilee, Israel; Division of Family Medicine, Rappaport Faculty of Medicine, Technion-Israel Institute of Technology, Haifa, Israel; The Cheryl Spencer Department of Nursing, University of Haifa, Haifa, Israel; Department of Ophthalmology, Rappaport Faculty of Medicine, Carmel Medical Center, The Technion-Israel Institute of Technology, Haifa, Israel

**Keywords:** Glaucoma, Primary care physicians, Physicians’ performance, Role perception

## Abstract

**Background:**

Glaucoma is a leading cause of blindness. The participation of primary care physicians (PCPs) in glaucoma care may improve health outcomes for glaucoma patients.

**Objectives:**

To investigate PCPs’ attitudes towards their role in glaucoma care, perceived barriers, and self-reported performance in glaucoma management.

**Methods:**

PCPs working in the Haifa and Western Galilee District of Clalit Health Services, Israel’s largest Health Maintenance Organization (HMO) were asked to complete a self-administered structured questionnaire. Physicians were asked to rate their agreement with statements describing the PCP’s role in glaucoma care, and to state how often they behave accordingly in their practice. In addition, physicians were asked to rate the extent that factors such as time constraints and knowledge gaps impede their performance in glaucoma care.

**Results:**

Eighty-two physicians completed the questionnaire. The majority thought that PCPs have a major role in early detection of glaucoma (99 %), discussing the importance of adherence to treatment (93 %), and encouraging patients to make regular visits to their ophthalmologist (99 %). However, only 30 % reported asking patients about family history of glaucoma, 64 % reported discussing adherence to treatment, and only 35 % stated that they explain how to use eye drops, while most of respondents (87 %) regularly provide refill prescriptions for glaucoma medications. Sixty percent claimed that during their residency they had not acquired adequate knowledge and competence to allow them to take proper care of glaucoma patients. The main barriers reported were lack of time (43 %), lack of knowledge regarding treatment options and recommended follow-up (46 %), and not being familiar with glaucoma medications’ side effects (54 %).

**Conclusions:**

There is a gap between PCPs’ perceptions of their role in glaucoma care and their report on actual performance in early detection and management of glaucoma. Further research is needed to develop and assess interventions that aim at closing this gap.

## Background

Primary open angle glaucoma (POAG), the most common type of glaucoma, is a leading cause of irreversible blindness, accounting for 2 % of visual impairment and 8 % of blindness globally [1]. In 2011, 2.71 million persons in the United States had POAG, with the highest estimated number among populations aged 70–79 years [2]. In the UK, the NHS recently reported more than one million glaucoma related visits per year [3]. In Israel, glaucoma affects approximately 10 % of persons aged 65 or above, and it is the third most common cause of blindness [4, 5].

Glaucoma is sometimes referred to as the “silent blinder” because of its asymptomatic nature until late in course. In developed countries, almost 70 % of cases are not detected [6], and 39 % of patients with glaucoma present with advanced disease in at least one eye [7]. Risk factors of family history, black race, advanced age and elevated intraocular pressure (IOP) are associated with increased risk for POAG [8]. Early diagnosis and treatment of glaucoma has been found clinically beneficial and cost effective, as it significantly delays visual field deterioration [9, 10]. Most patients receive IOP-lowering topical medications for the treatment of glaucoma and need lifelong treatment and regular follow-ups to improve outcomes [11]. However, adherence to glaucoma pharmacotherapy, defined as the extent to which a patient follows a treatment plan as it was prescribed [12], is often poor, as more than 25 % of glaucoma patients do not take their prescribed medications regularly [13]. Among the key reasons for this are patients’ lack of faith in the necessity of treatment and the fear of using eye drops [14, 15].

Primary care physicians (PCPs) are well positioned to participate in the prevention and management of glaucoma, by ensuring that at-risk patients are screened for the disease, and by providing educational information about the disease and its treatment. Patients perceive their PCPs as reliable and turn to them for advice and assistance on health-related issues in general and eye health in particular [16]. In a survey conducted in the USA by the National Eye Institute (NEI) almost all adults (96 %) responded that they would go for an eye checkup if their PCP recommended it [17]. Furthermore, the relationship between PCPs and their patients can influence patients’ health-related behavior such as adherence to medical treatment [18]. Additionally, good communication and collaboration between the PCP and the ophthalmologist in glaucoma care may assist in early detection of glaucoma medications’ systemic side effects, and in maintaining the continuity of visits to the ophthalmologist [19, 20].

In health care systems where the PCP is the patient’s case manager and provides monthly chronic prescriptions for all medical treatments, including ophthalmic medications [21], the PCP can ascertain the use of glaucoma medications as prescribed and detect barriers to adherence to glaucoma pharmacotherapy [22]. However, in our previous study, which explored factors associated with adherence to glaucoma pharmacotherapy among 738 patients with long-standing glaucoma, we found that PCPs, in general, are not involved in glaucoma care. Although the majority of the participants in that study (90 %) described their relationship with the PCP as good, only 18 % reported that the PCP ever discussed with them the importance of adherence to glaucoma treatment, and even fewer (16 %) claimed that their PCP had ever explained how to instil the eye drops [15].

In spite of the important role PCPs might have in glaucoma care, to the best of our knowledge, no study to date has explored factors that influence the delivery of glaucoma prevention and management by PCPs.

The current study aims to investigate PCPs’ attitudes towards their role in glaucoma care, self-reported performance in glaucoma management, and perceived barriers towards their participation in glaucoma prevention and treatment.

## Methods

### Participants

The study population consisted of PCPs registered in the 2011 computerized database of the Haifa and Western Galilee District of Clalit Health Services (CHS), Israel’s largest health maintenance organization (HMO), which has a well-established community care system and provides full medical coverage to over 50 % of Israel’s population, approximately 700,000 of whom reside in Haifa and its vicinity. All PCPs employed by Haifa and Western Galilee district of CHS (n = 380) are entitled to four fully paid weekly hours of continuing medical education (CME) activities funded by CHS. Physicians were approached during CME meetings in each of the five 2011-CME program venues. Upon agreement to participate in the survey, physicians were asked to complete an anonymous, structured, self-administered questionnaire and return it to the researchers by mail, fax or in person. The study was approved by the Clalit Health Services’ Institutional Review Board, at the Meier Medical Centre (No. K 027/2011). Written informed consent was obtained from all participants.

### Questionnaire

Based on a thorough literature review regarding the role of PCPs in glaucoma care [19, 20, 22–24], and potential barriers for the participation of PCPs in preventive care and risk factors management [25–27], a structured questionnaire was designed, consisting of the following sections:

- *Characteristics of the responding physicians* This section included personal data (i.e., age, gender, residency stage, medical school from which they graduated, number of years since MD graduation and glaucoma morbidity among the respondents and their first- degree relatives); and data on participants’ patient population (i.e., the number of patients aged 40 or above, and the number of glaucoma patients on their lists).
- *Self*-*reported performance in glaucoma care* Physicians were asked to rate on a numerical scale how often they behave in practice in accordance with statements describing the assumed role of the PCP in glaucoma care, including: early detection of glaucoma (1 item), management of glaucoma medical treatment (2 items) and adherence to treatment (2 items), and glaucoma care coordination and relationships with the ophthalmologist (3 items). Similarly, physicians were asked to rate how often they refill chronic prescription for glaucoma treatment (recommended by the ophthalmologist). The following response scale was used for all self- reported performance items: 1- never, 2- rarely, 3- sometimes, 4- often, 5- usually, 6-always.
- *PCPs’ attitudes and perceptions regarding their role in glaucoma care* Physicians were asked to rate to what extent they agree with each of the statements describing the assumed role of the PCP in glaucoma care using the following response scale: 1- not at all, 2- to a very small extent, 3- to a small extent, 4- to a moderate extent, 5- to a large extent, 6- to a very large extent.
- *PCPs’ perceived barriers to participation in glaucoma care* physicians were asked to rate on a numerical scale to what extent each of the following factors impedes their performance in glaucoma care: time, knowledge about glaucoma risk factors, knowledge regarding diagnostic evaluation, and knowledge regarding treatment of glaucoma and its adverse effects. Similarly, physicians were asked to rate to what extent, in their opinion, they had received adequate glaucoma-related education in medical school or during family medicine residency training. The following response scale was used for the above items: 1- not at all, 2- to a very small extent, 3- to a small extent, 4- to a moderate extent, 5- to a large extent, 6- to a very large extent.

The questionnaire was validated in the following manner: a multidisciplinary team consisting of two board certified PCPs (OCC, KK), and an ophthalmologist specializing in glaucoma care (OG) compiled a list of questions according to study objectives. The wording of the questions was further pre-tested by administering the questionnaire to a group of five board certified PCPs to ensure that the questions were intelligible and answerable. Physicians were debriefed after completing the questionnaire and the questionnaire was revised based on their comments.

### Data analysis

All data analyses were performed using the SPSS version 18 statistical program (SPSS INC, Chicago, IL, USA). Means and standard error deviations are reported for continuous variables. Pairwise comparison of means was based on a paired t test and comparison of percentages was based on χ^2^ test. Fisher’s exact test was used when cell frequencies were five or less. Correlations between categorical variables were examined using the Spearman’s rank correlation coefficient. Cronbach’s alpha was calculated to assess internal consistency of the items related to two domains: *self*-*reported performance in glaucoma care* (8 items); and *PCP’s perception of their role in glaucoma care* (8 items). The high level of internal consistency among items (Cronbach’s Alpha ≥0.8) enabled the formation of a new complex variable for each domain by calculating the mean score of the items of which it consists. A significant difference was defined as alpha <0.05 for all statistical tests.

## Results

Between February and December 2011, 167 PCPs, out of the 180 physicians (93 %) enrolled in the CME program, were invited to participate in the study. Of those invited, 82 (49 %) completed and returned the questionnaires. The respondents’ characteristics are presented in Table 1. Of the respondents included in the analyses, 19 (23 %) were residents in family medicine during hospital rotations, and reported that they could not estimate the number of glaucoma patients they were presently treating, from among all of their patients.

**Table 1:** Characteristics of study participants and their patients (n = 82)

Characteristics	
Age, years mean (SD)	43 (11), range 27–64
Gender, n (%)	
Men	31 (38)
Women	51 (62)
Years since medical school graduation, mean (SD)	15 (11), range 2–40
Place of medical school graduation, n (%)	
Israel	46 (56)
Former Soviet Union	16 (20)
Europe (excluding the Soviet Union)	15 (18)
Other	5 (6)
Glaucoma among participants (self report), n (%)	3 (4)
Glaucoma among family members, n (%)	10 (12)
Main place of work, n (%)	
Community clinic	63 (77)
Hospital	19 (23)
Estimated number of patients > 40 years old, n (%)	
<500	28 (34)
500≤	35 (43)
Don’t know	19 (23)
Estimated number of glaucoma patients, n (%)	
<20	34 (42)
20≤	29 (35)
Don’t know	19 (23)

Most of the PCPs who participated in this study (n = 71, 87 %) claimed that they often, usually, or always (i.e., answered 4, 5 or 6 on the numerical scale) provide monthly chronic prescriptions for glaucoma medications, and the majority of respondents (n = 76, 93 %) thought that the involvement of the PCP in glaucoma care can improve patients’ adherence to treatment.

Table 2 presents the rates of respondents who agree with statements describing the assumed role of the PCP in glaucoma care, side by side with the rates of respondents who report that they behave in practice according to each of these role descriptions.

**Table 2:** Primary care physicians’ self-reported performance in glaucoma care and agreement with role descriptions (n = 82)

Domain	Role description	Agree with the role description		Behave in practice accordingly	
		n (%)^b^	Mean (SD)	n (%)^a^	Mean (SD)
Early detection	Asking older patients about family history of glaucoma and referring them to complete ophthalmologic examination^1^/identifying patients at high risk of glaucoma and referring them to complete ophthalmologic examination^2^	81 (99)^2^	5.5 (0.8)^2^	24 (30)^1^	2.7 (1.5)^1^
Management of medical treatment	Explaining the proper use of eye drops to glaucoma patients	69 (85)	4.6 (1.2)	28 (35)	2.8 (1.4)
	Asking glaucoma patients about treatment’s adverse effects	71 (88)	5.1 (1.2)	37 (46)	3.3 (1.5)
Promoting adherence to treatment and follow up	Discussing the importance of adherence to treatment with glaucoma patients	75 (93)	5.2 (1.0)	52 (64)	4.0 (1.6)
	Encouraging glaucoma patients to have regular ophthalmologic follow-up examination	78 (99)	5.4 (0.7)	64 (81)	4.7 (1.4)
Coordination of eye care	Maintaining good relations with ophthalmologists working in the community clinics	74 (91)	5.1 (1.0)	45 (56)	3.6 (1.8)
	Informing the ophthalmologist on treatment’s adverse effects or contraindications to treatment	74 (93)	5.2 (1.0)	42 (53)	3.6 (1.5)
	Receiving and reading follow-up letters from the ophthalmologists concerning glaucoma patients	76 (94)	5.3 (1.0)	57 (70)	4.3 (1.5)

^a^Answered 4 or above on a 1–6 numerical scale (1-never; 6-always)

^b^Answered 4 or above on a 1–6 numerical scale (1-not at all; 6-to a very large extent)

^1^Statement describing PCP’s behavior in practice

^2^Statement describing the assumed role of the PCP in glaucoma care

Participants’ mean score for the extent of their agreement with each of the role descriptions ranged between 4.6 ± 1.2 (for “Explaining the proper use of eye drops to glaucoma patients”), and 5.5 ± 0.8 (for “Identifying patients at high risk of glaucoma”).

Participants’ mean score for the frequency of their performance according to each of the role descriptions ranged between 2.7 ± 1.5 (for “Asking older patients about family history of glaucoma”), and 4.7 ± 1.4 (for “Encouraging glaucoma patients to undergo periodic ophthalmologic follow-up examination”).

The mean score for the complex variable *self*-*reported performance in glaucoma care* (Cronbach’s alpha = 0.9) was significantly lower than the mean score for the complex variable *PCP’s perception of their role in glaucoma care* (Cronbach’s alpha = 0.8), (3.8 ± 1.1; 5.2 ± 0.6, respectively, p < 0.0001). However, a positive correlation was found between these two complex variables (r = 0.5, p < 0.001).

In addition, positive correlations were found between physicians’ age and number of years since MD graduation and *self*-*reported performance in glaucoma care* (r = 0.31, p = 0.007; r = 0.33, p = 0.005, respectively).

The barriers reported by the respondents for participating in glaucoma care are specified in Table 3. Negative correlations were found between physicians’ age and number of years since MD graduation, and the extent to which *lack of knowledge regarding glaucoma adverse effects* was perceived as a barrier to participate in glaucoma care (r = −0.29, p = 0.015; r = −0.24, p = 0.045, respectively). Similarly, a negative correlation was found between the number of years since MD graduation and the extent to which *lack of knowledge regarding glaucoma treatment and follow*-*up* was perceived as a barrier to participate in glaucoma care (r = −0.24, p = 0.05).

**Table 3:** Primary care physicians’ perceived barriers to participation in glaucoma care (n = 82)

Factors	n (%)^a^	Mean (SD)
Lack of time	36 (45)	3.1 (1.6)
Lack of knowledge regarding: Glaucoma risk factors Diagnostic evaluation Treatment options and recommended follow-up Glaucoma medications’ adverse effects	27 (33) 25 (30) 40 (48) 43 (53)	3.0 (1.5) 2.7 (1.6) 3.6 (1.3) 3.6 (1.1)

^a^Answered 4 and above on a 1–6 numerical scale (1-not at all; 6-to a very large extent)

In addition, most of the respondents (n = 57, 70 %) claimed that they had not been provided with adequate glaucoma-related knowledge during medical school, and more than half of the respondents (n = 49, 60 %) claimed that they had not acquired adequate glaucoma care-related knowledge and skills during their residency training.

## Discussion

The reported performance of PCPs who participated in this study varies greatly across the different domains of glaucoma care and is associated with physicians’ attitudes towards their role in the management of the disease. However, there is a significant gap between participants’ level of agreement with each of the role descriptions and the frequency of reported performance, which may imply that physicians in this study perceive their performance in glaucoma care as less-than-desirable. Most respondents reported that they do not usually ask patients about family history of glaucoma, although most of them agree that it is the role of the PCP to identify patients at high risk for glaucoma. Furthermore, although the majority of the PCPs provide refill authorizations for chronic, ophthalmologist-prescribed glaucoma prescriptions, many of them reported that they rarely explain how to use the drops to their patients, or inquire about glaucoma treatment’s adverse effects. On the other hand, physicians in this study reported being highly involved in promoting adherence to treatment and coordinating eye care. These behaviors are not glaucoma specific, and demand the application of the more “generic” skills of the PCP, which might make them self-evident and easier to perform. Indeed, study participants stated that glaucoma-related knowledge gaps, especially in relation to treatment’s adverse effects are major barriers to their performance in glaucoma care.

Other factors that were found to be associated in this study with physicians’ self-reported performance in glaucoma care were physicians’ age and their years of medical experience. One possible explanation is that older and more experienced physicians, (at least those who participate in CME programs), have managed to overcome glaucoma-related knowledge gaps that were not adequately attended to, according to study participants, during medical school or residency training. More experienced physicians might also deal better with time constraints, a topic which was also rated by many participants as a major barrier to their performance in glaucoma care.

This study is the first one, to our knowledge, to examine PCPs’ perception of their role in glaucoma care and their self-reported participation in the management of the disease. Our results conform to those of a recently published meta-ethnographic synthesis of qualitative research by Rubio-Valera et al., which found that factors affecting the performance of PCPs in the implementation of primary prevention and health promotion activities in adults include experience, skills and knowledge, as well as lack of time and the low importance ascribed to the subject matter in the medical school curriculum [28]. In addition, data from the 2012 American Academy of Family Physicians (AAFP) CME Needs Assessment survey also resembles the results of our current study by indicating that family physicians have knowledge gaps that may impede their optimal management of patients with glaucoma [29].

Our study has several limitations. The study was confined to a sample of PCPs attending a CME program, rather than all practicing PCPs. Hence, it is possible that the sample differed systematically from physicians who do not participate in a CME program. In addition, about a quarter of the sample (23 %, n = 19) were residents in family medicine. This and the relatively small sample size limit the generalizability of the findings. However, all the residents that participated in this study had already completed a 15-months rotation in community clinics working as full time PCPs. They were approached during in- hospital internal medicine or pediatric rotations, at the end of which they returned to the primary care clinics. Their glaucoma related educational needs and role perception could, but don’t necessarily reflect those of other young PCPs.

Physicians who agreed to participate in this study might have greater interest and more positive attitudes towards their role in glaucoma care compared to those who declined.

The high rates of participants’ agreement with the literature-based descriptions of their role in glaucoma care could be, at least in part, due to social desirability, and hence, possibly bias the study results. On the other hand, the application of this perspective to participants’ self-reported performance suggests that in reality, PCPs’ performance in glaucoma care is even more limited than reported in this study. Possible ways of encouraging PCPs to be more involved in glaucoma care are financial incentives and the use of reminders in computerized medical records, but the effectiveness of these tools is yet to be proven [28]. In addition, physicians can improve their care of glaucoma patients by engaging in continuing medical education that provides practical, current, evidence-based guidelines and recommendations related to glaucoma prevention and management [30].

## Conclusions

The results of the current study suggest that PCPs are highly aware of their role in glaucoma care; however, they often do not pursue this role, due to lack of knowledge and time.

Further research is needed to develop and assess interventions that address PCPs perceived barriers to participating in glaucoma care. Specifically, additional studies are needed to examine whether the participation of PCPs in a high-quality, outcome- based CME designed to address the educational needs described in this study improves their performance in glaucoma care.

## Abbreviations

PCPs: primary care physicians
POAG: primary open angle glaucoma
UK: United Kingdom
NHS: National Health Service
IOP: intraocular pressure
USA: United States of America
NEI: National Eye Institute
CHS: Clalit Health Services
HMO: Health Maintenance Organization
CME: continuing medical education
SD: standard deviation
MD: Medical Doctor
AAFP: American Academy of Family Physicians
